# Acknowledgement to Reviewers of *Pharmacy* in 2014

**DOI:** 10.3390/pharmacy3010001

**Published:** 2015-01-09

**Authors:** Pharmacy Editorial Office

**Affiliations:** MDPI AG, Klybeckstrasse 64, CH-4057 Basel, Switzerland

The editors of *Pharmacy* would like to express their sincere gratitude to the following reviewers for assessing manuscripts in 2014:

Alinier, Guillaume
Bíró, Éva
Boyd, Matthew
Cain, Jeff
Calcagno, A.
Cavé, Christian
Christiansen, Angela
Clapp, Alison
Denetclaw, Tina
Dhital, Ranjita
Dickson-Swift, Virginia A
Dipaula, Bethany A.
Divaris, Kimon
Fan, Mary D.
Fink, Joseph L.
Foisy, Michelle
Gallimore, Casey E.
Ghalili, Rose Ann DiMaria-
Grimes, Tamasine C.
Guilbert, Edith
Gustafsson, Lars L.
Hameem, Sakeena
Hanna, Lezley-Anne
Hughes, Christine
Hughes, Christine A
Hughes, Louise
Irie, Tetsumi
Jacobs, Sally
Jani, Yogini Hariprasad
Kearney, Kevin R.
Kidd, Emma
Knoderer, Chad
Kosa, Karolina
Kumpf, Vanessa
Lutz, Stephen
Maher, Judith H.
Maine, Lucinda
Malik, Muhammad
Matarese, Laura E.
McClelland, Gabrielle Tracy
McLaughlin, Deirdre
Meijerman, Irma
Moczygemba, Leticia R.
Morais, José
Payakachat, Nalin
Petrakaki, Dimitra
Price, Margaret
Quince, Thelma A.
Rathbun, R. Chris
Rekkas, Dimitrios
Ricci, Z.
Romanelli, Frank
Rouse, Mike
Rutter, Paul
Saito, Sayaka
Sealy, Patricia Ingrid
Shelton, Chasity M.
Simpson, Maree Donna
Skrobik, Yoanna
Spooner, Andy
Stitt, Danielle M.
Struble, Kimberly A.
Tran, Deanna
Tseng, Alice
Vermeulen, Lee C.
Warholak, Terri L.
Wiffen, Philip J.
Wilcock, Michael
Winslade, Nancy
Wright, David
Yu, Junhua
Yuksel, Nese

